# Real-World Clinical Characteristics and Outcomes with Daptomycin Use in Pediatric Patients: A Retrospective Case Series

**DOI:** 10.3390/antibiotics13090833

**Published:** 2024-09-02

**Authors:** Hanna Persha, Stephen A. Thacker, Krutika Mediwala Hornback, Gustavo R. Alvira-Arill, Richard Lueking, Taylor Morrisette

**Affiliations:** 1 Department of Pharmacy Services, Medical University of South Carolina Shawn Jenkins Children’s Hospital, Charleston, SC 29425, USA; persha@musc.edu (H.P.); alviraar@musc.edu (G.R.A.-A.); 2 Department of Pediatrics, Division of Infectious Diseases, Medical University of South Carolina Health, Charleston, SC 29425, USA; thackest@musc.edu; 3 Department of Pharmacy Services, Medical University of South Carolina, Charleston, SC 29425, USA; mediwala@musc.edu; 4 Department of Clinical Pharmacy & Outcomes Sciences, Medical University of South Carolina College of Pharmacy, Charleston, SC 29245, USA; 5 Department of Internal Medicine, Division of Infectious Diseases, Medical University of South Carolina Health, Charleston, SC 29425, USA; lueking@musc.edu

**Keywords:** adolescent, children, daptomycin, methicillin-resistant *Staphylococcus aureus*, pediatrics, vancomycin-resistant enterococci

## Abstract

Introduction: Daptomycin (DAP) is a cyclic lipopeptide that exhibits potent in vitro activity against many drug-resistant gram-positive organisms, including methicillin-resistant *Staphylococcus aureus* (MRSA) and vancomycin-resistant enterococci (VRE). Despite substantial reports evaluating the clinical outcomes of DAP within the adult population, real-world data are lacking in children. The primary goal of this evaluation was to describe the clinical characteristics and outcomes of DAP use in pediatric patients across a wide range of infections. Methods: This retrospective evaluation included patients < 18 years of age who were treated with DAP from January 2014 to May 2023. The primary objective was to evaluate the composite clinical success, which was defined as a 30-day survival, the lack of a 30-day microbiological recurrence, and the resolution of signs and symptoms of an acute infection without therapy modifications based on clinical failures. Secondary objectives included adverse effects potentially attributable to DAP and reasons for DAP utilization. Results: Forty patients were included, which were predominately male (62.5%) and white (52.5%), with a median age of 8.7 [IQR, 4.4–16.0] years. DAP was used for a wide range of infections, including central line-associated bloodstream infections (CLABSIs; 32.5%), infective endocarditis (15.0%), surgical-site infections (12.5%), and osteomyelitis (12.5%). The most common pathogen isolated was MRSA (37.5%), and most patients were bacteremic (60.0%). The median DAP dose was 8 [IQR, 6–10] mg/kg, and the median duration of the DAP therapy was 11.5 [IQR, 4.8–18.8] days. Most patients achieved composite clinical success (75.0%). An adverse effect occurred in 5.0% of the patients. DAP was prescribed the most for its ease of use/ability to facilitate discharge (40.0%) and/or for issues with alternative therapies (37.5%). Conclusion: Most pediatric patients that received DAP demonstrated clinical success with a low incidence of adverse effects. Larger, real-world studies of DAP use are necessary to further assess clinical outcomes.

## 1. Introduction

Infections caused by drug-resistant, gram-positive organisms in pediatric patients are increasing and are associated with substantial morbidity [1,2,3,4]. Specifically, methicillin-resistant *Staphylococcus* aureus (MRSA), methicillin-resistant coagulase-negative staphylococci (MR-CoNS), and vancomycin-resistant enterococci (VRE) are becoming a common cause of invasive infections in children [4]. Furthermore, these resistant organisms represent a threat due to the limited therapeutic options in neonates and other pediatric age groups in comparison to adults [4]. Unfortunately, it is common that data surrounding the use of many antimicrobials in children are not only lacking but also lagging those reported in adults [5,6]. Given the distinct scarcity of available literature related to both clinical success rates and the safety of newer antibiotics in pediatric patients, data evaluating the use of these agents are critical [4,6].

Daptomycin (DAP) is a cyclic lipopeptide that exhibits a rapid and concentration-dependent bactericidal activity against many drug-resistant gram-positive organisms, including MRSA, MR-CoNS, and VRE [7,8,9]. DAP is only currently Food and Drug Administration (FDA) approved in children aged one to seventeen years of age for the management of complicated skin and skin-structure infections (SSTIs) and *Staphylococcus aureus* bloodstream infections [7]. Importantly, DAP dosing in children varies based on age and indication, and higher doses are typically required to achieve target exposures due to an increased clearance in younger patients [7,10]. Abdel-Rahman and colleagues conducted a single-dose (DAP 4 mg/kg) pharmacokinetic analysis in children and found that estimates of the DAP total body exposure in adolescents were nearly 2× those observed in children that were less than six years of age [10]. Specifically, the mean area under the curve values were ~375 mcg × h/mL and ~215 mcg × h/mL in patients aged 12–17 years and 2–6 years, respectively [10]. As an example, this has, in part, led to DAP packaged dosing in children for SSTIs, with values in the range of 5–10 mg/kg every 24 h in comparison to 4 mg/kg every 24 h in adults [7].

Given its broad spectrum of activity against resistant gram-positive organisms, once-daily dosing in most pediatric patients with normal renal function, and its relatively simplistic monitoring parameters, DAP is an appealing therapeutic option in complicated pediatric infections [11]. However, data surrounding the real-world on- and off-label use of DAP in children and adolescents are lacking, and what data is published primarily originates from care outside of the United States, with notable differences in pathogen epidemiology and patient populations [12,13,14,15]. Therefore, the primary goal of this report was to evaluate the contemporary use, clinical characteristics, and outcomes of children treated with DAP.

## 2. Results

Overall, 40 patients were included in this evaluation. Seven patients were excluded as they were greater than 18 years of age at the time of DAP initiation, and two patients were excluded, as they had incomplete medical records that precluded the ability to extract all clinically relevant information. Patients were predominately white (21/40, 52.5%) and male (25/40, 62.5%), and the median [IQR] age of patients was 8.7 [4.4–16.0] years (Table 1). Overall, 10.0% of the patients were <1 year, 25.0% were between 1 and 5 years, 22.5% were between 6 and 12 years, and 42.5% were between 13 and <18 years. Most patients were admitted to an intensive care unit (ICU) (27/40, 67.5%). Most patients had at least one risk factor for MRSA infections, with the majority having been hospitalized within the prior 12 months (32/40, 80.0%) and/or having received antibiotics within 12 months (26/40, 65.0%). Overall, the median [IQR] hospital length of stay was 11.0 [7.0–32.0] days.

**Figure 1:**
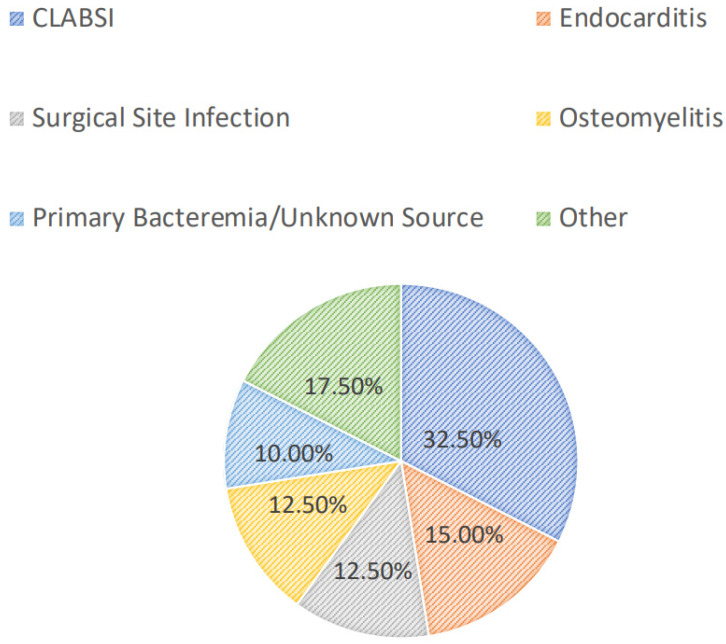
Infectious diagnoses. CLABSI: central line-associated bloodstream infection.

**Figure 2:**
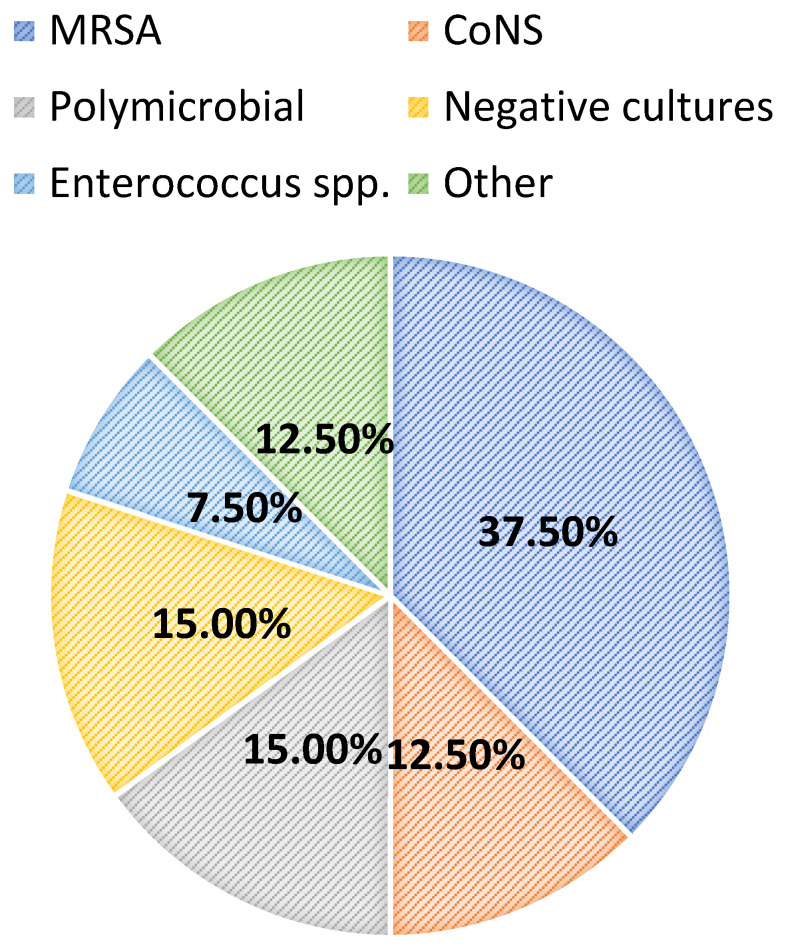
Microbiology data. MRSA: methicillin-resistant *Staphylococcus aureus;* CoNS: coagulase-negative staphylococci.

Most patients were diagnosed with a central line-associated bloodstream infection (13/40, 32.5%), followed by endocarditis (6/40, 15.0%), a surgical-site infection (5/40, 12.5%), osteomyelitis (5/40, 12.5%), or a primary bacteremia/unknown source (4/40, 10.0%). Among all patients, 24/40 (60.0%) had positive blood cultures. Overall, greater than half of the included patients had successful source-control procedures performed (21/40, 52.5%), with 33.3% (7/21) having an incision and drainage procedure, 14.3% (3/21) undergoing debridement, and 14.3% (3/21) undergoing central line or port removal. Furthermore, most patients (34/40, 85.0%) had organisms isolated from culture(s). Of these, the majority (15/34, 44.1%) grew MRSA as the sole organism, while 14.7% and 8.8% grew coagulase-negative staphylococci and *Enterococcus* spp., respectively. Polymicrobial cultures were observed in 6/34 (17.7%) of the cases. The infection diagnoses and microbiology data are shown in Figure 1 and Figure 2, respectively.

Nearly all patients (38/40, 95.0%) were on previous antibiotics prior to initiating DAP therapy, the most common of which were vancomycin (35/38, 92.1%), cefepime (17/38, 44.7%), ceftriaxone (13/38, 34.2%), and/or piperacillin-tazobactam (12/38, 31.6%). The median [IQR] duration of antibiotics received prior to DAP initiation was 4.2 [2.0–6.8] days. The vast majority of patients had a Pediatric Infectious Diseases consultation in place (37/40, 92.5%), and nearly all (36/40, 90.0%) were recommended to initiate or continue DAP by the Pediatric Infectious Diseases consultation service. The median [IQR] initial DAP dosing regimen was 8 [6,7,8,9,10] mg/kg, while the median [IQR] duration of the DAP therapy was 11.5 [4.8–18.8] days. Most patients (85.0%) received DAP for ≥72 h and were on concomitant antibiotics at some point during the DAP therapy (25/40, 62.5%). Importantly, 21/40 (52.5%) of the patients were prescribed DAP at discharge. All infection and treatment characteristics are shown in Table 2.

Most patients receiving DAP achieved composite clinical success (30/40, 75.0%). When breaking down the individual components of the composite primary outcome, 37/40 (92.5%) achieved survival at 30 days, 36/40 (90.0%) lacked microbiologic recurrence at 30 days, and 31/40 (77.5%) had a resolution of the signs and symptoms of acute infections while on the DAP therapy. Nearly one-third of the patients (12/40, 30.0%) were re-admitted within 30 days of discharge. Of the patients that failed therapy (n = 10), 60.0% were bacteremic, and of these patients that had organisms isolated (n = 7), 28.6% grew MRSA, and 14.3% grew VRE.

**Figure 3:**
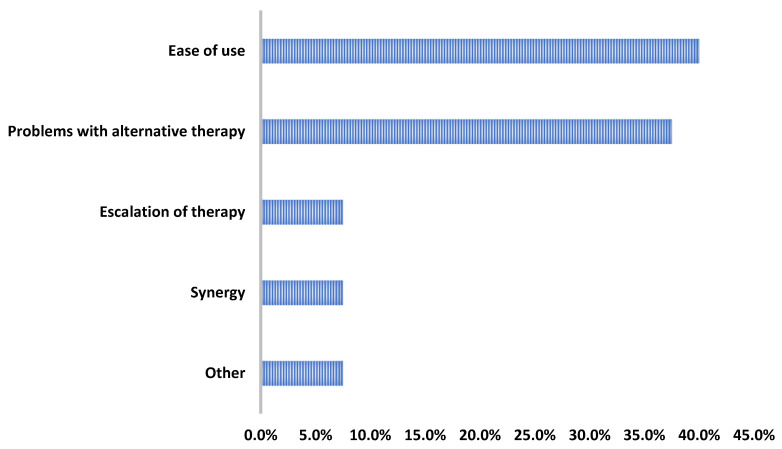
Reasons for daptomycin use.

Overall, only two patients experienced adverse effects: one patient had an elevated creatine phosphokinase that resolved (baseline CPK: 62 U/L; peak CPK: 572 U/L; last CPK while still on DAP: 98 U/L) and another patient that had excessive nighttime sweating that began once starting DAP. Regarding the provider rationale for use, the DAP therapy was most commonly initiated for its ease of use and/or its ability to facilitate discharge (16/40, 40%) or due to barriers with alternative therapies (15/40, 37.5%) (Figure 3).

## 3. Discussion

This retrospective, real-world evaluation of DAP use in pediatric patients demonstrated utilization for a wide variety of pathogens and infection types with high rates of clinical success and low rates of adverse effects. Furthermore, this is one of the largest reviews of on- and off-label contemporary DAP utilization in pediatric patients from the United States thus far. It further provides the overall trends and clinical characteristics of the use of DAP in pediatrics. Due to the in vitro potency against drug-resistant gram-positive organisms and the once-daily dosing regimen in most children, DAP is an appealing option to use in many inpatient and outpatient clinical scenarios [7,8].

Vancomycin is the gold-standard parenteral therapy for invasive gram-positive infections in both adults and children [16,17]. The popularity of vancomycin is largely due its long-standing historical precedent, enhanced provider comfortability, low prevalence of resistance for many commonly encountered gram-positive organisms, and the fact that no other agent has yet to show superiority in randomized controlled trials (RCTs) [16,17]. However, vancomycin has several drawbacks: the need for therapeutic drug monitoring in most infections (and an inadequate consensus on how to most optimally perform vancomycin monitoring in children), the high risk of nephrotoxicity, the relatively slow bactericidal activity, and frequent dosing intervals that are typically required in children due to rapid renal clearance [16,18]. Linezolid, another commonly used option for resistant gram-positive infections, is limited by side effects associated with long-term use, certain provider concerns due to its bacteriostatic activity, theoretical drug–drug interactions with serotonergic agents, and the thrice-daily dosing interval in pediatric patients < 12 years of age [19,20,21].

Daptomycin has been evaluated in multiple multicenter, comparator RCTs in pediatric patients aged one to 17 years [22,23]. Bradley and colleagues prospectively evaluated the efficacy and safety of DAP versus the standard of care (2:1 ratio; primarily vancomycin or clindamycin) in children with complicated SSTIs that were suspected or confirmed to be caused by gram-positive organisms [22]. Overall, 257 DAP and 132 standard-of-care patients were included. Within the DAP group, 44.8% and 37.1% of patients had confirmed MRSA or methicillin-susceptible *Staphylococcus aureus*, respectively. The clinical success rates 7–14 days after the last dose of the antibiotic were similar for both groups (87.0–91.0%), and the most common adverse effects in the DAP group were diarrhea (7.0%) and/or increased creatine phosphokinase (5.5%) [22].

Another study conducted by Arrieta and colleagues randomized pediatric patients with suspected or proven *S. aureus* bacteremia at a 2:1 ratio to receive daptomycin versus the standard of care (primarily vancomycin or cefazolin) [23]. Overall, 55 children were randomized to DAP and 27 children to the standard of care, with the most common infectious sources in the DAP group being primary bacteremia/an unknown source or an intra-abdominal infection (21.8% each) or a catheter-related infection, SSTI, or bone infection (20.0%) each. The most common pathogens isolated were methicillin-susceptible *S. aureus* (80.0%) and MRSA (12.7%) within the DAP group. Clinical response rates at the test-of-cure visit were similar for both groups (DAP group: 88.2% vs. standard-of-care group: 77.3%) and were also relatively similar across each age cohort, and the most common side effects in the DAP group included diarrhea, increased creatine phosphokinase, and increased hepatic enzymes (3.8% each) [23].

In comparison to RCTs, real-world studies are extremely important for front-line clinicians, as they provide information with respect to instrumental health outcomes regarding the use of drugs in specific conditions, patient populations, and organisms beyond those that are typically included in RCTs. Although there have been other studies evaluating the real-world use of DAP in the pediatric population, nearly all of these have been conducted in areas outside of the United States, with differences in pathogen epidemiology and patient populations [12,13,14,15]. Vonasek and colleagues conducted a retrospective evaluation of children that received at least one dose of DAP [15]. A majority of the patients were between 5–12 years of age (38.8%) and received DAP for bacteremia, including CLABSIs (29.3%), osteomyelitis or septic arthritis (25.9%), or SSTIs (25.9%). Throughout the cohort, only 10.9% of the patients experienced clinical worsening or a lack of improvement while on DAP, while common side effects included new-onset loose or watery stools (9.5%), increased AST/ALT (6.1–8.8%), or increased creatine phosphokinase (2.0%) [15]. Overall, the results of our evaluation mirror the limited, more contemporary data that exist for DAP use in pediatric patients [15]. Although other studies have evaluated DAP use within the United States, they are limited in that they provide data from over a decade ago and not all reports include clinical outcomes [24,25,26].

The dosing of DAP in the pediatric population varies based on age, as younger patients have an increased DAP clearance and require higher doses to achieve target exposures [10]. In the adult population, DAP is FDA-approved at a dosage regimen of 4–6 mg/kg; however, the use of DAP doses between 8–12 mg/kg have been associated with enhanced in vitro and in vivo pharmacodynamic activity, preventing subsequent resistance and improved health outcomes in patients with invasive gram-positive infections [7,9,27,28,29,30,31]. Importantly, the DAP packaged label dosing for children is based on PK data which exhibit similar exposures to those seen in the adult population receiving 4–6 mg/kg of DAP once daily [10,22,23,32,33].

As such, Olney and colleagues aimed to assess DAP exposures achieved with pediatric packaged label dosing and to identify potential dosing regimens required to enhance efficacy in children with *S. aureus* bacteremia via Monte Carlo simulations for various pediatric age cohorts [34]. The pediatric packaged label dosing for DAP yielded insufficient exposures, with the probability of target attainment for efficacy hitting only 26.3% in children 13–24 months, 39.5% in children 2–6 years of age, 30.1% in children 7–11 years of age, and 50.1% in adolescents ≥ 12 years of age (goal is ≥90.0%). Based on this PK/PD analysis, DAP doses higher than what is currently within the pediatric package labeling would be required to target validated DAP efficacy targets [34].

Within our analysis, a high clinical success rate was observed even though most patients did receive packaged label dosing. However, not all patients were being treated for *S. aureus* bacteremia, and of the patients that experienced clinical failures, the most isolated organism was MRSA. Furthermore, within the PK/PD analysis conducted by Olney and colleagues, it is important to note that the minimum inhibitory concentration (MIC) utilized within the pharmacodynamic analysis was fixed at 1 mg/L (the current breakpoint for *S. aureus* of the Clinical and Laboratory Standards Institute) in the simulations that yielded insufficient exposures [34,35]. Although MIC data were not collected in our evaluation, it is presumed that most of the MRSA isolates had MIC values less than 1 mg/L, as the DAP MIC_90_ (MIC required to inhibit 90% of isolates) has been reported to be 0.5 mg/L [36]. Furthermore, DAP was initiated following a median [IQR] of 4.2 [2.0–6.8] days of previous antibiotic therapy. Given that the previous antibiotics likely reduced the overall inoculum of infection, higher success rates with potentially non-optimized doses are sensible, especially in cases in which the source control was pursued. Studies evaluating the efficacy and safety of higher DAP doses for MRSA infections in children are warranted.

The major limitation of this report is the retrospective nature of this study with no comparator group. Furthermore, patients could have been readmitted to another facility outside of our healthcare system with recurrence or worsening while on therapy, which could falsely hyperinflate our observed clinical success rates. Also, there was no minimum duration of DAP required for inclusion within this study, which could also hyperinflate the clinical success rates being truly attributable to the DAP in patients with shorter durations of the DAP therapy. Importantly, however, 85.0% of the patients received DAP for at least 72 h. Additionally, susceptibility data for DAP were rarely reported and thus were not included within our analysis. However, DAP non-susceptibility throughout the United States is relatively low in most gram-positive isolates, especially from those isolated from the pediatric population [36]. Finally, to our knowledge, there were no major changes in the international guideline recommendations for DAP use within the pediatric population that occurred within our study time frame. However, it is important to note that the lack of local institutional guidance for DAP use resulted in a wide variety of doses and indications, but our report does reflect the real-world use of DAP based on the discretion of front-line providers at the bedside.

## 4. Methods

This was a retrospective evaluation of DAP utilization conducted at the Medical University of South Carolina (MUSC) Health. Pediatric patients less than 18 years of age who received DAP at the Children’s Hospital between January 2014 and May 2023 were included. Patients were excluded if they were transferred from outside hospitals, and if there was an inability to collect all necessary data collection variables from medical records. Baseline demographics, clinical and treatment characteristics, clinical outcomes, and provider rationale for utilizing DAP were extracted from the electronic medical records.

The primary objective was to evaluate rates of composite clinical success, which was defined as survival at 30 days, the resolution of signs and symptoms of acute infection without therapy modifications based on clinical failures, and the lack of a 30-day microbiologic recurrence. The component of the primary composite outcome involving alterations in therapy did not include patients with modifications of their antimicrobial regimen for de-escalation purposes and/or for a step-down therapy. Secondary objectives included adverse effects potentially attributable to DAP and provider rationale for using DAP.

The data were analyzed utilizing descriptive statistics with nominal data reported as raw numbers and percentages and continuous data reported as medians [interquartile range, IQR]. Statistical analyses were performed using the IBM SPSS software, version 29.0 (SPSS, Inc., Chicago, IL, USA) and Microsoft Excel, version 16.77.1 (Redmond, WA, USA). The protocol for this study was submitted to the local institutional review board and was determined to be exempt from a full board review.

## 5. Conclusions

The primary objective of our report was to evaluate the contemporary use, clinical characteristics, and outcomes of DAP within the pediatric population. The high rates of clinical success and relative safety of DAP use across a wide array of pediatric age groups, infections, and pathogens provides further data supporting the claim that DAP may be a reasonable option for children when clinically appropriate. Additional real-world use data paired with multicenter trials are needed to define optimal indications of DAP use in pediatrics.

## Figures and Tables

**Table 1:** Baseline and clinical characteristics.

	N = 40
Male	25 (62.5%)
Age: years, median (IQR)	9 (4–16)
White	21 (52.5%)
Black	16 (40.0%)
ICU admission	27 (67.5%)
PICU	16 (40.0%)
PCICU	11 (27.5%)
MRSA risk factors	--
-Hospitalization within 12 months	32 (80.0%)
-Antibiotic exposure within 12 months	26 (65.0%)
-Invasive procedures within 12 months	18 (45.0%)
-Long-term central venous access within 12 months	14 (35.0%)
-Prior ABSSSI within 12 months	1 (2.5%)
Source control	21 (52.5%)
-Incision and drainage	7 (33.0%)
-Debridement	3 (14.3%)
-Central line or port removal	3 (14.3%)
Duration of hospitalization: days, median (IQR)	11 (7–32)
Baseline CPK: units/L, median (IQR)	60 (27–102) *
Initial temperature: degrees Celsius, median (IQR)	37.2 (36.8–38.3)
Initial heart rate: beats per minute, median (IQR)	115 (103–141)
Initial respiratory rate: breaths per minute, median (IQR)	23 (20–31)
Initial white blood cell count: median (IQR)	11.9 (6.4–20.9) **
Initial CRP: mg/dL, median (IQR)	7.6 (3.2–13.3) ***
Initial procalcitonin: ng/mL, median (IQR)	0.6 (0.2–8.6) ****
Initial serum creatinine: mg/dL, median (IQR)	0.6 (0.3–0.7) *****

Results are reported as n (%), unless otherwise noted. * n = 35, ** n = 33, *** n = 20, **** n = 13, and ***** n = 37. IQR: interquartile range; ICU: intensive care unit; PICU: pediatric intensive care unit; PCICU: pediatric cardiac intensive care unit; MRSA: methicillin-resistant *Staphylococcus aureus*; ABSSSI: acute bacterial skin and skin-structure infection; CPK: creatine phosphokinase; CRP: C-reactive protein.

**Table 2:** Infection and treatment characteristics.

	Results (n = 40)
Pediatric ID consult	37 (92.5%)
DAP recommended by pediatric ID	36 (90.0%)
Antibiotics prior to DAP initiation	38 (95.0%)
Total duration of antibiotics prior to DAP initiation: days, median (IQR)	4 (2–7)
DAP dosing: mg/kg, median (IQR)	8 (6–10)
Length of DAP duration: days, median (IQR)	12 (5–19)
Concomitant antibiotics	25 (62.5%)
DAP at discharge	21 (52.5%)

Results are reported as n (%), unless otherwise noted. ID: infectious disease; DAP: daptomycin.

## Data Availability

Data can be available upon request.
